# Combined Activity of Colloid Nanosilver and *Zataria Multiﬂora Boiss* Essential Oil-Mechanism of Action and Biofilm Removal Activity

**DOI:** 10.15171/apb.2017.074

**Published:** 2017-12-31

**Authors:** Maryam Shirdel, Hossein Tajik, Mehran Moradi

**Affiliations:** ^1^Department of Food Hygiene and Quality Control, Faculty of Veterinary Medicine, Urmia University, 1177, Urmia, West Azarbaijan, Iran.; ^2^Department of Medicinal and Industrial Plant, Institute of Biotechnology, Urmia University, 1177 Urmia, Iran.

**Keywords:** Antimicrobial, Sanitizing, Biofilm, Essential oil, Silver nanoparticle, Combination

## Abstract

***Purpose:*** The aim of this study was to investigate antimicrobial and biofilm removal potential of Zataria multiflora essential oil (ZEO) and silver nanoparticle (SNP) alone and in combination on Staphylococcus aureus and Salmonella Typhimurium and evaluate the mechanism of action.

***Methods:*** The minimum inhibitory concentration (MIC), and optimal inhibitory combination (OIC) of ZEO and SNP were determined according to fractional inhibitory concentration (FIC) method. Biofilm removal potential and leakage pattern of 260-nm absorbing material from the bacterial cell during exposure to the compounds were also investigated.

***Results:*** MICs of SNP for both bacteria were the same as 25 μg/ mL. The MICs and MBCs values of ZEO were 2500 and 1250 μg/mL, respectively. The most effective OIC value for SNP and ZEO against Salm. Typhimurium and Staph. aureus were 12.5, 625 and 0.78, 1250 μg/ mL, respectively. ZEO and SNP at MIC and OIC concentrations represented a strong removal ability (>70%) on biofilm. Moreover, ZEO at MIC and OIC concentrations did a 6-log reduction of primary inoculated bacteria during 15 min contact time. The effect of ZEO on the loss of 260-nm material from the cell was faster than SNP during 15 and 60 min.

***Conclusion:*** Combination of ZEO and SNP had significant sanitizing activity on examined bacteria which may be suitable for disinfecting the surfaces.

## Introduction

Nanotechnology, the science of study and use of structures at nanoscale, is a promising tool for producing novel materials with biomedical applications. Silver nanoparticles (SNPs) has been extensively investigated in various scientific disciplines. Silver in the form of silver nitrate and silver sulfadiazine has been widely used to cure bacterial infections associated with burns. SNPs are considered as a potent antimicrobial, antifungal, antiviral and antiprotozoal compound and it is also reported to have anti-inflammatory activities.^1^ Synthesis and use of SNPs as a new generation of antimicrobial agents would be a fascinating and economical tool to solve drug resistance. Microorganisms display different responses to nanoparticles which is related to differences in the bacterial structure and the composition of the cell wall.^1,2^

Essential oils (EOs), as a plant secondary metabolites, are volatile aromatic compounds extracted from different parts of plants. For centuries, EOs have been used in medicine, perfumery, cosmetics and food. They are primarily used in medicine, but in the nineteenth-century, EOs have found their importance to impart aroma and taste ingredients.^3^ Lamiaceae is a family of plant with more than 230 genera, which distributed nearly worldwide. It contains many well- known species with fairly similar properties in botanical characteristics and applications, including *Thymus vulgaris*, *Thymus caramanicus*, *Zataria multiflora*, *Ziziphora clinopodioides* and *Ziziphora tenuior.*^3-5^*Zataria multiflora* Boiss is an important medicinal plant, distributed in Iran, Afghanistan, and Pakistan. The main antimicrobial compounds of *Zataria multiﬂora* Boiss essential oil (ZEO) are thymol, carvacrol, and p-cymene. The plant also contains tannins, flavonoids, saponins and some bitter substances.^6^ Among the phenolic compounds, thymol is the most characteristic chemical substance of ZEO which founds in leaves, flowers, and roots at various amounts.^6,7^ ZEO displays inhibitory activity on both gram-negative and gram-positive bacteria.^8^

The practical advantage of antimicrobial combinations has been comprehended for over 50 years. Combinations of two antimicrobial agents with a different mechanism of action, may enhance antimicrobial activity especially where resistance to a single agent develops by bacteria. Also, due to synergy or additive interaction, the combination of drugs allows utilizing lower antimicrobial concentration, reducing the harmful side effects and increasing treatment efficacy.^9^ Tackling public health issues occur by the growing number of multidrug-resistant bacteria, proposes new antimicrobial formulation based on the combination of older antimicrobials with a rich source of new agents, such as natural products.^10^ Simultaneous use of EOs and other antimicrobial compounds with great disinfectant properties has a high priority since using EOs in high concentrations make some unpleasant organoleptic changes in food and also on food contact surface when used as a sanitizing compound. Owing to the possible synergistic properties, combined use of EO with SNPs as a sanitizing mixture for food plant sanitation has been proposed.^11^ The combined application of nanoparticles with EO has been reported.^2,12^ To understand the combined antimicrobial and biofilm removal properties of SNP and ZEO, their effects on two important bacterial pathogens, *Staph aureus* and *Salm.* Typhimurium was investigated as a guideline for a possible application of formulated solution in sanitation schedule.

## Materials and Methods

Silver nanoparticles solution (4000 µg/ mL with the particle size of 35 nm) was purchased from Pars Nano Nasb Co (Tehran, Iran) and sterilized by filtration through 0.22 µm filters before use. Peptone water, phosphate buffered saline (PBS), Luria-Bertani (LB) broth and agar, Agar-agar, and resazurin sodium were obtained from Sigma Chemical Co (St. Louis, MO., USA). All other chemicals were purchased from Merck (Darmstadt, Germany). The plant, *Zataria multiflora* Boiss, was purchased from local groceries. *Staph. aureus* ATCC 25923 and *Salm.* Typhimurium ATCC 14028 were obtained from the Department of Food Hygiene and Quality Control, Urmia University, Urmia, Iran.

### Preparation of bacterial suspension 

*Staph. aureus* and *Salm.* Typhimurium were grown at 37±1 °C for 18 h by transferring 10 microliters of frozen stocks (at -20 °C) into 10 mL of LB broth. Bacterial suspensions were adjusted to ~8 log_10_ CFU mL^-1^ using visible-ultraviolet spectrophotometer (Amersham Pharmacia Biotech Inc., Buckinghamshire, UK) at 600 nm (optical density: ~0.1) and confirmed by plating and counting on LB agar after 24 h incubation at 37±1 °C.

### Essential oil extraction and quantiﬁcation

The EO of *Zataria multiflora* Boiss (100 g) was extracted from dried aerial parts of plant using a Clevenger apparatus based on hydrodistillation procedure for 3 hours. The collected ZEO was dried over anhydrous Na_2_SO_4_, then filtrated and stored at 4 °C. The chemical composition of ZEOs was analyzed using a gas chromatograph (GC), as explained previously.^6^

### Determination of Minimum inhibitory concentration (MIC) and minimum bactericidal concentrations (MBC) 

The MICs and MBC of ZEO and SNP against both bacteria were determined in LB using broth microdilution assay in 96-wells polystyrene flat-bottomed microtitre plates based on CLSI guidelines.^13^ Two-fold serial dilutions of ZEO were prepared in 0.1% peptone water containing 0.15% agar (to make a stable emulsion of EO in peptone water), whereas dilutions of SNP were made in 0.1% peptone water. The wells of a microplates with U-bottom were poured by 160 μL of LB and 20 μL of the bacterial suspension with OD_600_ = 0.1 to reach a suspension with 10^6^ CFU/ mL in each well. Then, 20 μL either ZEO or SNP concentrations were added into the desired wells to achieve final concentrations of 312 to 5000 μg/ mL for ZEO and 62.5 to 2000 μg/mL for SNP. For every experiment, three controls, including LB alone, LB with bacteria and LB containing treatment agents without bacteria were used. The plate was mixed on a plate shaker at 250 rpm for 20 s and incubated at 37 ± 1 °C for 24 h. MICs were determined visually and by using 0.01% (w/v) resazurin sodium salt solution as explained previously. To determine MBCs, 10 µL from each well was inoculated into LB agar at 37 ± 1 °C for 24 h. The MBC was determined as the lowest concentration of antimicrobial agents that produces 99.99% inhibition in the initial population of microorganism. MBC: MIC ratio, which describes the relationship between the minimum *in vitro* bactericidal concentration and the MIC of antimicrobial agents, were also investigated.

### Antimicrobial combination and interaction

Broth checkerboard micro-assay was carried out to evaluate the antagonistic, indifferent, additive and synergistic interactions of ZEO and SNP using the fractional inhibitory concentration (FIC) index method.^14^ Eight different concentrations, 5 concentrations lower than MIC, two concentrations higher than MIC and one concentration as same as MIC of ZEO and SNP were used to design an 8 × 8 checkerboards of combinations. The microplates were prepared by dispensing 160 μL of LB and 20 μL of the logarithmic suspension of bacteria and 10 μL of different concentration of both antimicrobial agents into each well. Then, plates were kept in a plate shaker at 250 rpm for 20 s and incubated at 37 ± 1 °C for 24 h. MICs were determined visually and by resazurin reduction. MICs of the individual antimicrobials and all of the combinations were used to calculate Fractional inhibitory concentrations (FICs) of ZEO and SNP and FIC index using the following formula:

$$FIC\;of\;antibacterial\frac{MIC\;of\;antibacterial\;in\;combination}{MIC\;of\;antibacterial\;alone}$$

$$FIC\;index\;\left(FICI\right)\;=\;FIC_{ZEO}+\;FIC_{SNP}$$

If the FICI is < 0.5, the interaction is synergistic, if the FICI= 0.5-1, the interaction is additive, if the FICI= 1-4, the interaction is indifferent, and an FICI >4 is considered antagonistic. Optimal inhibitory combinations (OIC) is defined as the combinations producing an inhibitory effect by using the lowest concentration of one antimicrobial in combination with the other.^15^

### Determination of the release of 260-nm absorbing material

260-nm absorbing material released into the supernatant was estimated according to the method described previously,^16^ with some modifications. The bacterial suspension was prepared as described above. The procedure performed in 2 mL of harvested and washed cells (OD_600nm_ = 0.45) to which SNP and ZEO were added at final concentrations equivalent to their MICs and OIC and incubated in a shaker incubator (250 rpm at 37 °C). Two samples were taken at 15 and 60 min time points and centrifuged at 4000 *g* for 15 min and the absorbance was determined at 260 nm using PBS as blank. Controls containing bacterial supernatant without treatment agents and antibacterial compounds without bacteria were also prepared and analyzed.

### Biofilm removal

Biofilm removal potential of both agents alone and in combination was assessed using 24-well flat-bottomed polystyrene microtiter plates according to the method explained previously with some modifications.^17^ An aliquot of 200 μL of bacterial suspension with OD_600_ = 0.1 was dispensed into each well which was filled previously with 1800 μL of LB broth, using four repetitions per treatment to reach a suspension with 10^7^ CFU/ mL per well. After incubation for 24 h at 37 ±1 °C, the planktonic cells in wells were then removed and the plates were washed three times with PBS and air-dried for 20 min at 23 ±2 °C. Then, 2000 μL of MIC, 2MIC and 4MIC concentrations of ZEO and SNP and their combinations (1/4 OIC, 1/2OIC, and OIC concentrations) were gently poured into the wells and incubated for 15 min at ambient temperature. The solutions were then removed and plates were washed three times with PBS and air-dried for 20 min at 23 ±2 °C. Following staining with 2 mL of 1% crystal violet (CV) (w/v) for 30 min, the contents of the wells were decanted and washed twice with tap water to remove the color excess and then allowed to air-dry for 30 min. The biomass of biofilms was quantified by solubilizing CV with 2 mL of acetic acid 33% and subsequent measuring optical absorbance (OD) at 540 nm. Wells containing LB broth and LB with bacteria without antibacterial were considered as negative and positive controls, respectively. Biofilm removal percentage was calculated as follow:

$$Reduction\;percentage=\frac{\left(C-B\right)-\left(T-B\right)}{\left(C-B\right)}\times 100$$

where C is OD_540nm_ of control wells, B is OD_540nm_ of negative controls and T is OD_540nm_ of treated wells.

### Combined sanitizing activity 

Sanitizing effects of ZEO and SNP combination were investigated by determining the growth of the microorganism in LB broth supplemented with different concentration of antimicrobials (1/4OIC, 1/2OIC, and OIC) to obtain optimal concentration for both compounds according to the method explained before with some modifications.^18^ Bacterial cultures (1×10^7^ CFU/ mL) (0.5 mL) were added to tubes containing 4.5 mL of antimicrobial agents in combination and then, tubes were incubated at 23± 2 °C for 15 min and bacterial growth was monitored by sampling (1mL) and counting the viable cells. After sampling, 1 mL aliquot were dispersed in a 9 mL neutralizing solution containing an equal volume of sodium thiosulphate 5% w/v and Tween-20 and remained for 10 min, to neutralize the subsequent activity of agents and then serial dilutions were prepared.

### Statistical analysis

Each experiment was replicated in triplicate and carried out on three separate times and data were expressed as means ± S.E. Data were analyzed by analysis of variance (ANOVA, *P*<0.05) using GraphPad Prism version 5.00 for Windows, GraphPad Software, San Diego California USA (www.graphpad.com).

## Results

### ZEO chemical analysis

As shown in Table 1, 26 different components were identified in ZEO, representing 97.23%, of total EO. General chemical profile of ZEO was characterized by an abundance concentration of thymol (44%). In addition to thymol, carvacrol (14.04%) and *p*-cymene (11.15%), as main constituents, and traces of linalool, ϒ-terpinene, and α-Pinene were also found in ZEO.

### Antibacterial properties of ZEO and SNP 

The inhibitory effects of ZEO and SNP alone and in combination against *Staph. aureus* and *Salm*. Typhimurium were investigated using microtiter plate assay. For *Staph. aureus*, the MIC values of ZEO and SNP were 1250 and 25 μg/mL and for *Salm*. Typhimurium the values were 2500 and 25 μg/mL, respectively. In all cases, MBC values were similar to MICs. The ZEO was found to be more effective on gram-positive than gram-negative bacteria whereas SNP displayed similar antibacterial activity on both bacteria. The MICs for SNP - ZEO combination were 0.78 and 12.5 μg/ mL against *Staph. aureus* and *Salm*. Typhimurium, respectively. ZEO-SNP combination inhibited *S. aureus* and *Salm*. Typhimurium at 625 μg/ mL. Based on the FICI scale (Table 2), the combination displayed a synergistic action on *Staph. aureus* (FICI=0.81) and *Salm*. Typhimurium (FICI= 0.75).

### Loss of 260 nm absorbing material

**Figure 1:**
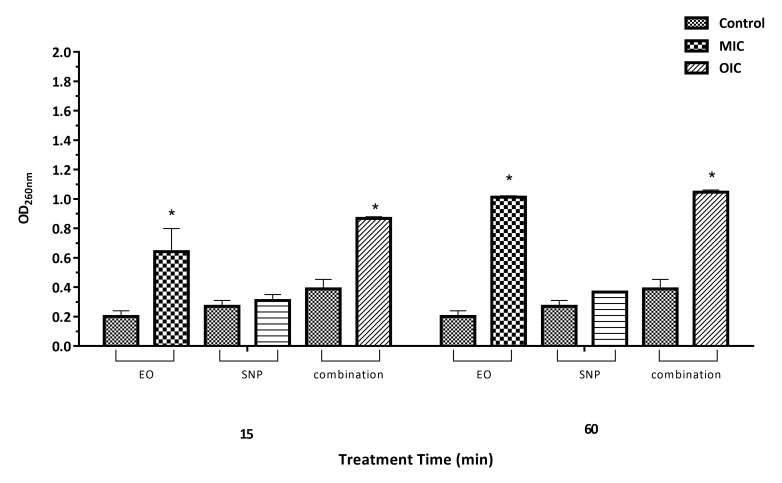
260-nm absorbing material released from *Staph. aureus* cells after treatment with MIC concentration of silver nanoparticle (SNP), *Zataria multiflora* essential oil (EO) and their combination. Asterisks indicate significantly different values (*P*<0.05) when comparing optical density (OD) of control and each treatment at the same exposure time.

**Figure 2:**
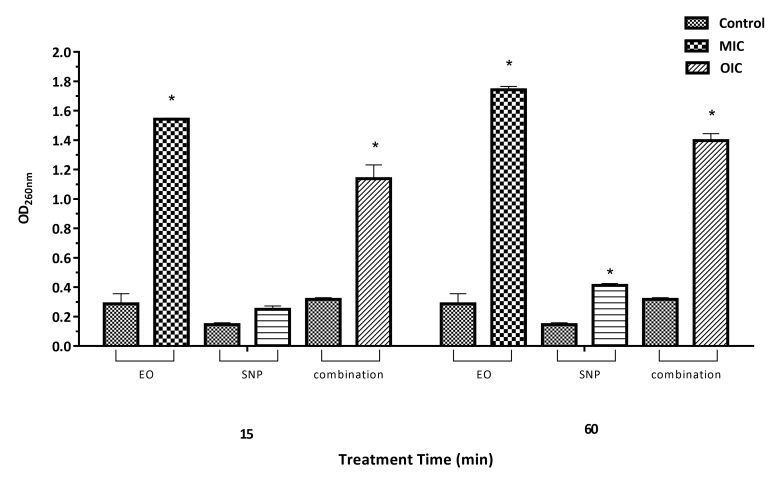
260-nm absorbing material released from *Salm.* Typhimurium cells after treatment with MIC concentration of silver nanoparticle (SNP), *Zataria multiflora* essential oil (EO) and their combination. Asterisks indicate significantly different values (*P*<0.05) when comparing optical density (OD) of control and each treatment at the same exposure time.

Exposure of *Staph. aureus* and *Salm*. Typhimurium to ZEO at MIC concentrations over 15 min resulted in a significant increase in loss of 260 nm absorbing material from the bacterial cell compared with the control (*P*<0.05), indicating a stronger disruption of the cell membrane by ZEO (Figure 1, 2). Results also showed that the loss began before 15 min and continued up to 60 min. In general, loss of cytoplasmic materials by ZEO from both bacteria was faster than SNP. The leakage pattern of 260-nm absorbing material was directly linked to the sanitizing activity of both antimicrobial compounds. As shown in Figures 1 and 2, no significant release of 260 nm absorbing material with SNP- treated at MIC concentration over 15 min for both bacteria was found. Higher exposure time (60 min) significantly increased the cell leakage of *Salm*. Typhimurium, but not in case of *Staph. aureus.* Longer exposures (60 min) with OIC concentration of ZEO and SNP (Figures 1 and 2), caused 3 and 4-fold more gross membrane damage, in *Staph. aureus* and *Salm*. Typhimurium culture compared to the control sample, respectively.

**Table 1:** Chemical composition of ZEO.

**Compounds**	**KI** ^a^	**Area (%)**	**Compounds**	**KI**	**Area (%)**
α-Thujene	931	0.15	linalool	1106	6.26
α-Pinene	639	3.66	Borneol	1163	0.19
Camphene	953	0.17	Terpinen-4-ol	1175	4.63
β-Pinene	980	1.55	Thymol methyl ether	1233	0.17
β-Mycrene	991	1.35	Bornyl acetate	1284	0.17
α-Phellandrene	1002	0.15	Thymol	1301	44
d-3-Carene	1009	1	Carvacrol	1318	14.4
α-Terpinene	1016	1.74	Acetylthymol	1284	0.3
Cis-para-menth-2-en-1-o	1123	0.37	d-Elemene	1340	0.24
p-Cymene	1028	11.15	Eugenol	1360	0.82
1,8-Cineole	1032	0.86	*Trans*-Caryophyllene	1423	2.78
γ-Terpinene	1060	0.08	Caryophyllene oxide	1583	0.93
α-Terpinolene	1087	0.08			
**Total**					97.23%

^a^Kovats indices calculated against *n*-alkanes on HP-5 column.

**Table 2:** Survival population (log CFU/ mL) of *Staph. aureus* and *Salm.* Typhimurium treated with ZEO and SNP alone and in combination during 15 min contact time at room temperature.

Microorganism*	**Alone at MIC concentration**		**In combination**		
	**ZEO**	**SNP**	**OIC**	**1/2OIC**	**1/4OIC**
*Staph. aureus*	0	3.11 × 10^5^± 0.08^a^	0	3.11 × 10^5^± 0.67^a^	4.74 × 10^5^± 0.41^a^
*Salm.* Typhimurium	0	4.69 × 10^5^± 0.19^b^	0	3.90 × 10^5^± 0.21^b^	5.30 × 10^5^± 0.33^b^

*Initial bacterial counts: 10^6^ CFU mL^-1^. Different letters for each column of indicate a statistically significant difference (*P* < 0.05).

### Biofilm removal

**Figure 3:**
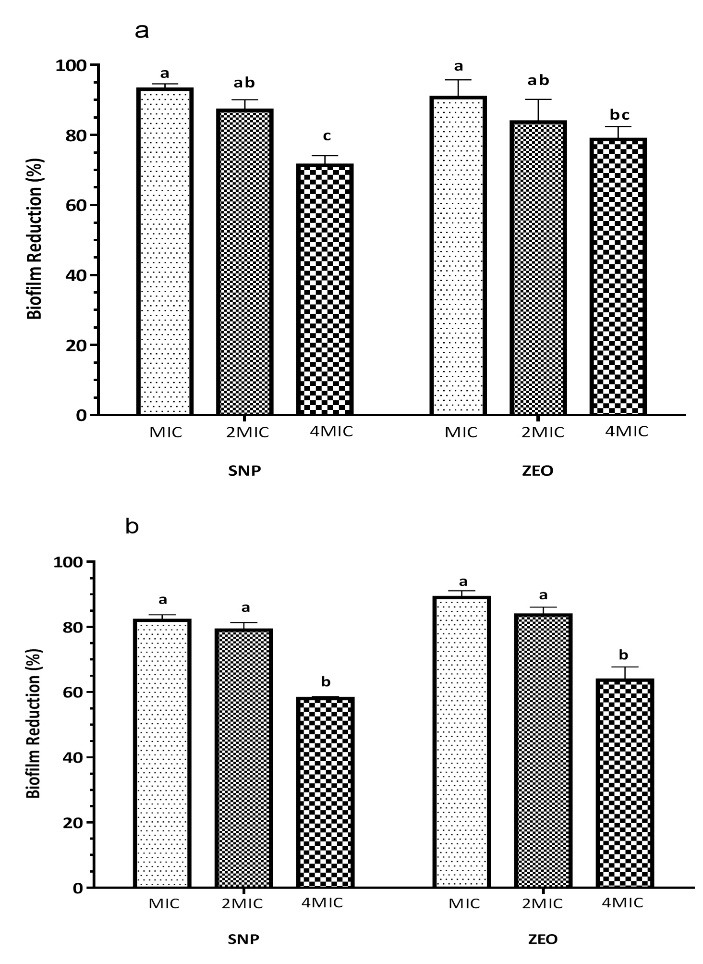
The effect of silver nanoparticle (SNP) and *Zataria multiflora* essential oil (ZEO) on removal one-day old biofilm of *Staph. aureus* (**a**) and *Salm.* Typhimurium (**b**) developed on polystyrene surface with 15 min contact time. Different letters for each concentration of antimicrobial indicate a statistically significant difference (*P* < 0.05).

As compared with *Salm.* Typhimurium (Figure 3b), higher removal of *Staph. aureus* biofilms were achieved at 15 min exposure time for SNP-ZEO (Figure 3a). Results demonstrated that biofilm of *Staph. aureus* is more sensitive to SNP - ZEO than *Salm*. Typhimurium. SNP at MIC, 2MIC, and 4MIC concentrations resulted in 93, 87 and 71.33% and 82, 79 and 58% removal of *Staph. aureus* and *Salm. Typhimurium* cells, respectively. For* Staph. aureus* and *Salm*. Typhimurium, 90.6%, and 89% bacteria were eliminated at a concentration of MIC for ZEO. Concerning the hydrophobicity property of ZEO, it can be observed in Figure 3, a reduction in the biofilm removal with increasing ZEO concentration supporting the use of surfactants could help in the disruption of such shortcoming.

### Synergistic sanitizing activity 

The purpose of this experiment was to achieve a best effective concentration of SNP and ZEO as a sanitizing solution. To assess the disinfectants efficiencies, MIC and OIC concentrations were determined. The reductions in the *Staph*. *aureus* and *Salm*. Typhimurium counts after 15 min exposure to MIC and various OIC concentrations were shown in Table 2. A reduction of 100% was achieved for both bacteria after 15 min exposure to ZEO. A similar reduction was also found for OIC concentration. Whereas, less than 0.4 log_10_ CFU/ mL reduction was observed for the samples treated with SNP in alone.

As shown by 260 nm absorbing material measurements, SNP displayed an antimicrobial activity after a long time of exposure (at least after 1 h) compared with ZEO. In comparison with *Staph. aureus*, *Salm*. Typhimurium had higher resistance to SNP. Additionally, no significant reduction (*P* < 0.05) was observed in the microbial counts of 1/2OIC and 1/4OIC with SNP treated samples.

## Discussion

The components identified in ZEO (Table 1) in this study was similar to previous works. According to Saedi Dezaki et al. (2007),^19^ the major components of ZEO were thymol (41.81%), carvacrol (28.85%), and *p*-cymene (5.63-13.16%). In the study of Saei-Dehkordi, et al. (2010),^7^ a variation of the major components, thymol (27.5-64.87%), carvacrol (2.7-22.39), *p*-cymene (8.36%) and linalool (0-7.92%) of ZEO were observed, due to the collection of plant material from five main phytogeographic grown towns in Iran. Factors such as growth phase and geographic origin of plant, method of plant drying and processing affect EO content and chemical compositions.^7^

In our study, the antibacterial properties of ZEO was not different to the results reported from other studies, apart from some detected variable bacterial susceptibilities that may cause the differences in the chemical composition of ZEOs and the bacterial strains used by others. The hydrophobic property of most EOs could increase their permeability and accumulation in the bacterial cell membranes.^6,7^

Silver ions and silver-based compounds are widely known as highly toxic to 16 major species of bacteria due to their multiple mechanisms of action. Zarei et al. (2014) reported the MIC values of SNP against *Listeria monocytogenes*,* S.* Typhimurium, *Escherichia coli* O157:H7 and *Vibrio parahaemolyticus* in the range of 3.12- 6.25 µg mL^-1^ according to microdilution method in Tryptic soy broth.^20^ The small size and high ratio of surface to volume in SNPs, allow them to more effectively contact with microorganisms and induce their antimicrobial activities.^21^ It has been shown that antibacterial efficacy of SNP varies between different prepared SNPs. Given the fact that factors such as the type of bacteria, the media of inoculation, type and the size of nanoparticle and the method of preparation must be considered when comparing the antibacterial activity of different nanoparticles.^1^

The additive or partial synergy effect is a type of interaction in which the combined effect is equal to the sum of the effects of the individual agents. As shown previously,^22^ SNP interaction with EO is a bacteria-dependent phenomenon. The synergistic properties of SNP with ZEO on *Staph. epidermidis* and *Staph. aureus* (FICI= 0.5-1) has been demonstrated, but no synergistic effects were found against Methicillin-resistant *Staph. aureus* and *Ps. aeruginosa* (FICI value of 3 and 1.25, respectively).

The MBC: MIC ratio ≥ 8 is considered as an indicator of bacteriostatic activity.^23^ In the current study, MIC/MBC of both SNP and ZEO were 1:1, suggesting a bactericidal effect on *Staph. aureus* and *Salm. Typhimurium*. It was demonstrated that oregano EO reveals a synergistic activity with common antibiotics such as gentamicin against some potential pathogens,^24^ additive activity in combination with another antibiotic such as amoxicillin and polymyxin on Extended-Spectrum Beta-Lactamase (ESBL)-producing *E.coli,*^25^ and synergistic with other EO obtained from *Thymus vulgare* and *Rosmarinus officinalis*.^26^

Measuring the loss of 260-nm absorbing material from the bacteria is an indicator of cell leakage which could be used to understand the mechanism of action of antimicrobial compounds. The presence of materials with an absorbance at 260 nm in the supernatant of bacterial solution indicates a loss of nucleic acids from the cell. Based on the results of this study (Figures 1 and 2), the higher values determined by the measurements at 260 nm are an indication for the leakage of bacterial contents which subsequently confirms the physical damages of bacterial cell walls by ZEO.^4^ However, the reductions in the efficacy of SNP may be best explained by bacterial blocking caused by higher concentrations of SNP, which could reduce the contact surface of nanoparticles with bacteria and its antibacterial efficacy.

SNP can cause agglomeration after adding to the nutritious media such as LB and as shown previously,^2^ treatment of *Staph. aureus* with a combination of EO and SNP, induced a reduction in cell density, exopolysaccharide, morphology changes, and cell destruction. However, membrane permeability created by EO might allow the small molecules of SNP to enter the cell.

**Figure 4:**
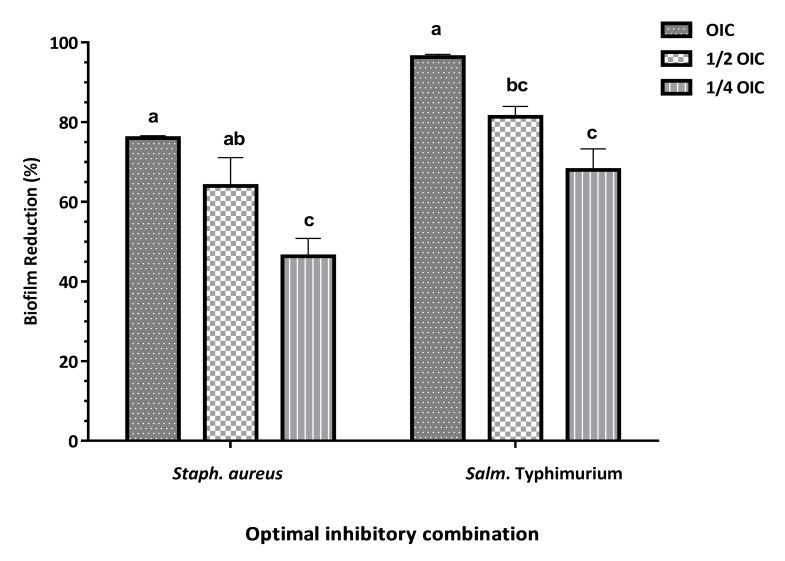
The effect of silver nanoparticle (SNP) and *Zataria multiflora* essential oil (ZEO) combination on removal one-day old biofilm of *Staph. aureus* and *Salm.* Typhimurium developed on polystyrene surface with 15 min contact time. Different letters for each concentration of antimicrobial indicate a statistically significant difference (*P* < 0.05).

Researchers are aware of the importance of biofilms in causing diseases and drug resistance, therefore finding a safe and effective method for biofilm removal is of great importance. Natural agents are considered as a safe way to remove biofilms. Although the research on SNP interaction with biofilm is still in its early phases, we currently know that removal of biofilm by SNP could occur in three steps: transportation to the vicinity of the biofilm, attachment, and penetration within the biofilm.^27^ It has been shown that eradication of biofilms by SNP is not a concentration-dependent process, rather it occurs in a time-dependent manner.^28^ The main compounds of ZEO are thymol, a monoterpene phenolic derivative, and its phenol isomer, carvacrol. Both of them contribute to antimicrobial and antioxidant properties of Lamiaceae family.^4^ Additionally, hydrophobic characteristics of these ingredients allow the penetration of ZEO into outer exopolysaccharide and inner layers of biofilms.^29^ In our study, by decreasing the OIC concentration from 1/2OIC to 1/4OIC (Figure 4), the biofilm removal properties were significantly decreased (*P*<0.05) from 64 % to 43.33 % for *Staph. aureus* (Figure 4) whereas OIC concentration, removed 76% of biofilm mass. For *Salm.* Typhimurium (Figure 4) the values were 96.46%, 81.50% and 68 % for OIC, 1/2OIC and 1/4OIC concentrations, respectively. It means that combined use of SNP and ZEO boosted biofilm removal potential of both antibacterial compounds against different pathogens. According to Gurunathan et al. (2014), the combined use of NPs and antibiotic such as ampicillin exhibit antibiofilm activity on Gram-positive and Gram-negative bacteria by 55 and 70%, whereas combining those NPs with vancomycin revealed a 75 and 55% reduction of biofilm of Gram-positive and Gram-negative bacteria.^1^ It is worth mentioning that, differences in the method used to evaluate biofilm removal activity and differences in the sensitivity of different bacteria, the age of biofilm and type of surface which biofilm developed could cause different results in the biofilm removal percentage in previous studies.

## Conclusion

The results of our study demonstrated that SNP and ZEO have synergistic antibacterial activities against *Staph*. *aureus,* and *Salm*. Typhimurium. It was also shown that the antimicrobial and biofilm removal properties of SNP and ZEO were affected by the type of microorganisms and concentrations of both compounds. ZEO displayed a fast antimicrobial activity. Both antimicrobials represented considerable biofilm removal activity on both bacteria. The combination of SNP and ZEO was additive, which means significant antibacterial and antibiofilm activity could achieve by use of agents at concentrations without compromising their antibacterial effects. The best concentrations for SNP- ZEO sanitizing solution were 12.5 μg/ mL for SNP and 625 μg/ mL for ZEO. Our results highlighted the powerful combination activity of SNP and ZEO which accelerated antibacterial activity, alleviated undesirable sensorial property of ZEO and reduced the concentration of both compounds.

## Acknowledgments

This study was funded by a grant from Faculty of Veterinary Medicine and Institute of Biotechnology, Urmia University. The authors would like to thank Dr. Mahmoudian for his assistance.

## Ethical Issues

Not applicable.

## Conflict of Interest

The authors declare no conflict of interest related to this work.
